# Synthesis and Evaluation of Novel ^68^Ga-Labeled [D-Phe^6^,Leu^13^ψThz^14^]bombesin(6-14) Analogs for Cancer Imaging with Positron Emission Tomography

**DOI:** 10.3390/ph17050621

**Published:** 2024-05-11

**Authors:** Lei Wang, Chao-Cheng Chen, Zhengxing Zhang, Hsiou-Ting Kuo, Chengcheng Zhang, Nadine Colpo, Helen Merkens, François Bénard, Kuo-Shyan Lin

**Affiliations:** 1 Department of Molecular Oncology, BC Cancer Research Institute, Vancouver, BC V5Z 1L3, Canada; lewang@bccrc.ca (L.W.); ccchen@bccrc.ca (C.-C.C.); zx1981ok@hotmail.com (Z.Z.); htkuo0325@gmail.com (H.-T.K.); cczhang@a9oncology.com (C.Z.); ncolpo@bccrc.ca (N.C.); hmerkens@bccrc.ca (H.M.); fbenard@bccrc.ca (F.B.); 2 Department of Molecular Imaging and Therapy, BC Cancer, Vancouver, BC V5Z 4E6, Canada; 3 Department of Radiology, University of British Columbia, Vancouver, BC V5Z 1M9, Canada

**Keywords:** gastrin-releasing peptide receptor, antagonist, positron emission tomography, Gallium-68, pancreas uptake

## Abstract

Gastrin-releasing peptide receptor (GRPR) is overexpressed in various cancers and is a promising target for cancer diagnosis and therapy. However, the high pancreas uptake and/or metabolic instability observed for most reported GRPR-targeted radioligands might limit their clinical applications. Our group recently reported a GRPR-targeted antagonist tracer, [^68^Ga]Ga-TacsBOMB2 ([^68^Ga]Ga-DOTA-Pip-D-Phe^6^-Gln^7^-Trp^8^-Ala^9^-Val^10^-Gly^11^-His^12^-Leu^13^ψThz^14^-NH_2_), which showed a minimal pancreas uptake in a preclinical mouse model. In this study, we synthesized four derivatives with unnatural amino acid substitutions (Tle^10^-derived Ga-LW01158, NMe-His^12^-derived Ga-LW01160, α-Me-Trp^8^- and Tle^10^-derived Ga-LW01186, and Tle^10^- and N-Me-Gly^11^-derived Ga-LW02002) and evaluated their potential for detecting GRPR-expressing tumors with positron emission tomography (PET). The binding affinities (K_i_(GRPR)) of Ga-LW01158, Ga-LW01160, Ga-LW01186, and Ga-LW02002 were 5.11 ± 0.47, 187 ± 17.8, 6.94 ± 0.95, and 11.0 ± 0.39 nM, respectively. [^68^Ga]Ga-LW01158, [^68^Ga]Ga-LW01186, and [^68^Ga]Ga-LW02002 enabled clear visualization of subcutaneously implanted human prostate cancer PC-3 tumor xenografts in mice in PET images. Ex vivo biodistribution studies showed that [^68^Ga]Ga-LW01158 had the highest tumor uptake (11.2 ± 0.65 %ID/g) and good tumor-to-background uptake ratios at 1 h post-injection. Comparable in vivo stabilities were observed for [^68^Ga]Ga-LW01158, [^68^Ga]Ga-LW01186, and [^68^Ga]Ga-LW02002 (76.5–80.7% remaining intact in mouse plasma at 15 min post-injection). In summary, the Tle^10^ substitution, either alone or combined with α-Me-Trp^8^ or NMe-Gly^11^ substitution, in Ga-TacsBOMB2 generates derivatives that retained good GRPR binding affinity and in vivo stability. With good tumor uptake and tumor-to-background imaging contrast, [^68^Ga]Ga-LW01158 is promising for detecting GRPR-expressing lesions with PET.

## 1. Introduction

As a member of the transmembrane G protein-coupled receptors, gastrin-releasing peptide receptor (GRPR) is expressed in the pancreas, gastrointestinal tract, and central nervous system, and it regulates a series of physiological functions such as hormone secretion, smooth muscle contraction, and synaptic plasticity [1,2,3]. Moreover, GRPR is overexpressed in a variety of malignancies, including breast, prostate, lung, and colon cancers, and the activation of GRPR leads to the proliferation of cancer cells [4,5,6,7]. Thus, GRPR is considered a promising target for the design of targeted radiopharmaceuticals for the diagnosis and radioligand therapy of GRPR-expressing cancers.

Bombesin (BBN), isolated from the skin of the European frog, *Bombina bombina*, is a natural exogenous ligand with a good binding affinity toward GRPR. The heptapeptide sequence at the C-terminus (bombesin(8-14)) is the minimal sequence needed for binding to GRPR with a high affinity. Thus, this peptide sequence has been used for the design of GRPR-targeted radiopharmaceuticals for cancer diagnosis and radioligand therapy [8,9,10,11,12,13,14,15]. Although several GRPR-targeted radiotracers have been evaluated in the clinic, the extraordinarily high pancreas uptake might limit the detection of pancreatic cancer and the metastatic lesions of other cancers in and/or adjacent to the pancreas. In addition, to avoid damage to the pancreas, the maximum tolerated dose might have to be lowered, and this could potentially lead to a suboptimal treatment efficacy for radiotherapeutic application [9,13,14,16].

**Figure 1:**
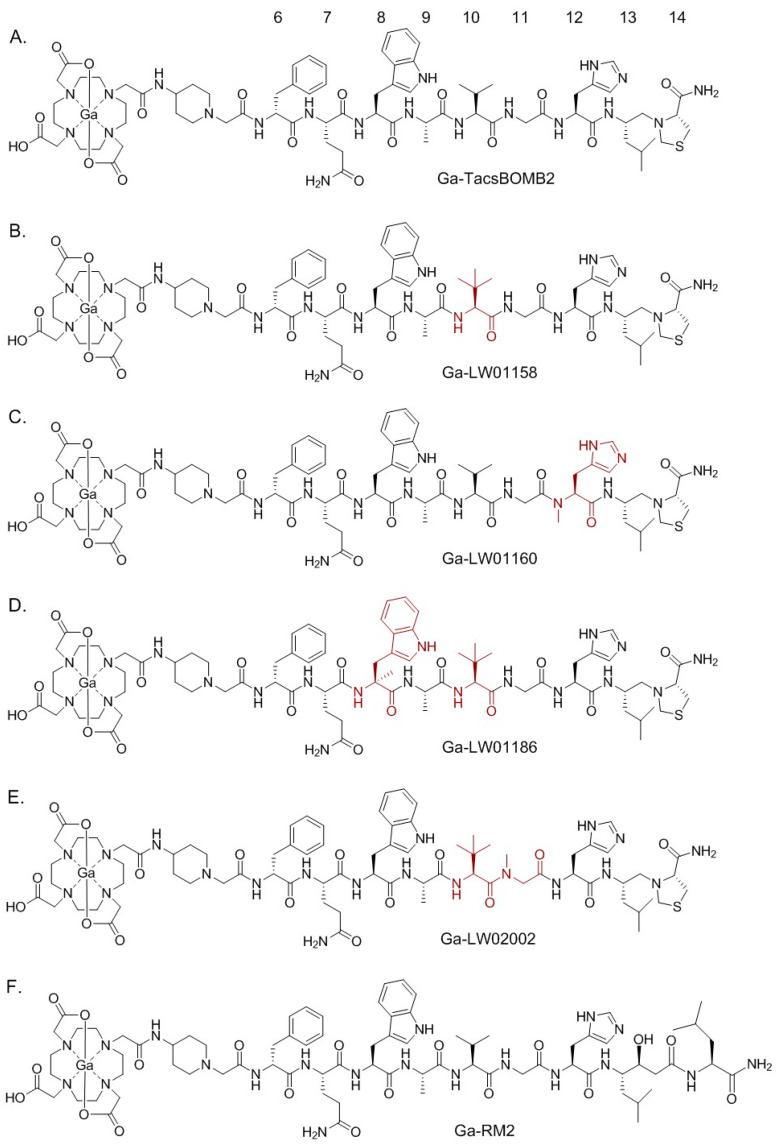
Chemical structures of (**A**) Ga-TacsBOMB2, (**B**) Ga-LW01158, (**C**) Ga-LW01160, (**D**) Ga-LW01186, (**E**) Ga-LW02002, and (**F**) Ga-RM2. The unnatural amino acid substitutions in Ga-TacsBOMB2 derivatives are shown in brown.

Inspired by the potent GRPR antagonist, RC-3950-II ([D-Phe^6^, Leu^13^ψThz^14^]Bombesin(6-14)), reported by the Schally group [17,18], our group synthesized and evaluated a ^68^Ga-labeled DOTA-conjugated RC-3950-II derivative, [^68^Ga]Ga-TacsBOMB2 (Figure 1A), for imaging GRPR-expressing cancer with positron emission tomography (PET) [19]. The GRPR antagonist characteristics of Ga-TacsBOMB2 were confirmed via intracellular calcium release assay. The potent GRPR binding affinity (K_i_) of Ga-TacsBOMB2 at a low nM scale contributes to the good uptake of [^68^Ga]Ga-TacsBOMB2 in human prostate cancer PC-3 tumor xenografts (10.2 ± 2.27%ID/g) at 1 h post-injection. Most importantly, the pancreas uptake value of [^68^Ga]Ga-TacsBOMB2 (2.81 ± 0.78%ID/g) was much lower than that of the clinically validated GRPR tracer, [^68^Ga]Ga-RM2 (41.9 ± 10.1%ID/g), in the same preclinical animal model [19].

Similar to most of the reported GRPR-targeted ligands, Ga-TacsBOMB2 could potentially be enzymatically degraded in vivo, especially by the neutral endopeptidase 24.11 (NEP, EC 3.4.24.11, neprilysin) [20,21]. The amide bonds between Gln^7^-Trp^8^, Trp^8^-Ala^9^, Ala^9^-Val^10^, and His^12^-Leu^13^ have been identified as the cleavage sites of clinically validated GRPR-targeted radioligands derived from RM2 and AMBA [10,22]. In this study, we hypothesized that (1) the amide bonds between Gln^7^-Trp^8^, Trp^8^-Ala^9^, Ala^9^-Val^10^, and His^12^-Leu^13^ in Ga-TacsBOMB2 (Figure 1A) are also potential cleavage sites of peptidases, and (2) replacing the amino acids adjacent to the potential cleavage sites in Ga-TacsBOMB2 with a closely related unnatural amino acid could improve its vivo stability and potentially retain a high GRPR binding affinity and low pancreas uptake characteristics.

Hence, in this study, we synthesized Ga-labeled LW01158, LW01160, LW01186, and LW02002 (Figure 1B–E) by replacing the natural amino acids adjacent to the cleavage sites with a closely related unnatural amino acid. We determined their antagonist/agonist characteristics with an in vitro fluorescence-based calcium release assay. The potential of these ligands for detecting GRPR-expressing cancer was evaluated by an in vitro competition binding assay, PET imaging, and ex vivo biodistribution studies in PC-3 tumor-bearing mice. The biodistribution data of these novel tracers were compared with previously reported data on [^68^Ga]Ga-RM2 (Figure 1F) obtained using the same preclinical tumor model [19].

## 2. Results

### 2.1. Syntheses of GRPR-Targeted Ligands

The yields for the synthesis of LW01158, LW01160, LW01186, and LW02002 ranged from 8 to 32%, and the yields for the synthesis of their nonradioactive Ga-complexed standards ranged from 76 to 81% (Tables S1 and S2). The identities of all precursors and nonradioactive Ga-complexed standards were confirmed by MS analyses (Tables S1 and S2 and Figures S1–S8). ^68^Ga-labeled LW01158, LW01186, and LW02002 were purified by HPLC and obtained in 16–61% decay-corrected radiochemical yields with 132–298 GBq/µmol molar activity and >92% radiochemical purity (Table S3).

### 2.2. Binding Affinity, Antagonist Characterization, and Hydrophilicity

**Figure 2:**
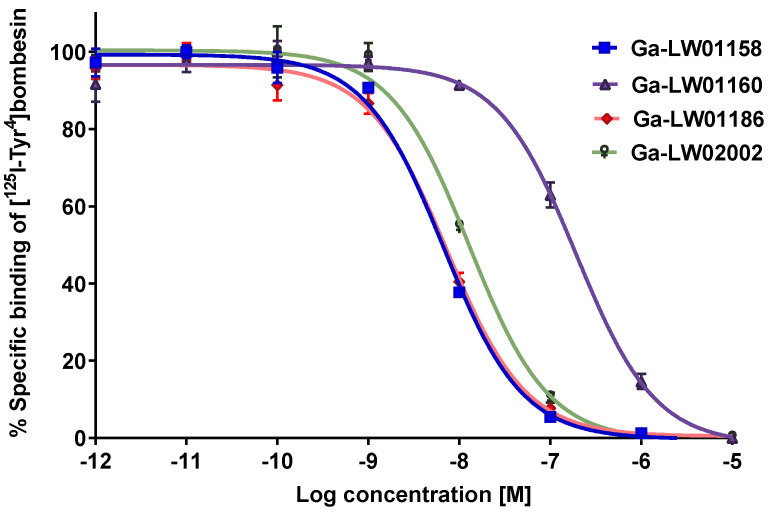
Displacement curves of [^125^I-Tyr^4^]Bombesin caused by Ga-LW01158, Ga-LW01160, Ga-LW01186, and Ga-LW02002 generated using GRPR-expressing PC-3 cells. Error bars indicate standard deviation.

As shown in Figure 2, the binding of [^125^I-Tyr^4^]Bombesin to PC-3 cells was inhibited by Ga-LW01158, Ga-LW01160, Ga-LW01186, and Ga-LW02002 in a dose-dependent manner. The calculated K_i_ values for Ga-LW01158, Ga-LW01160, Ga-LW01186, and Ga-LW02002 were 5.11 ± 0.47, 187 ± 17.8, 6.94 ± 0.95, and 11.0 ± 0.39 nM, respectively (n = 3).

**Figure 3:**
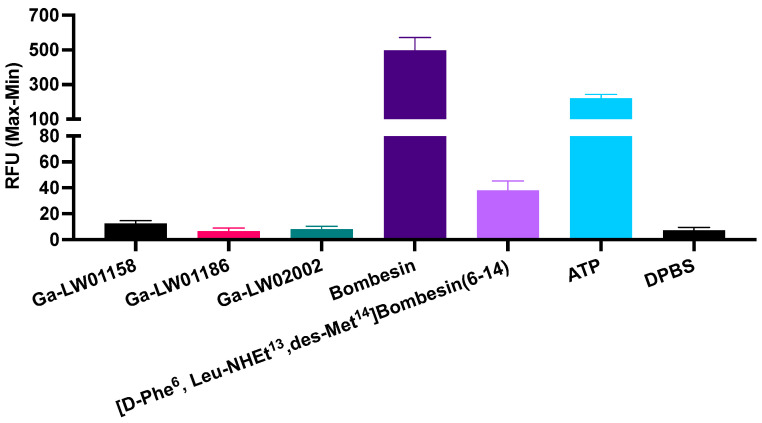
Intracellular calcium efflux in PC-3 cells induced by Ga-LW01158, Ga-LW01186, Ga-LW02002, bombesin, ([D-Phe^6^,Leu-NHEt^13^,des-Met^14^]Bombesin(6-14), ATP, and DPBS. Error bars indicate standard deviation (n = 3).

Since Ga-LW01160 had a poor GRPR binding affinity (K_i_ = 187 ± 17.8 nM), next we determined the agonist/antagonist characteristics only for the potent Ga-LW01158, Ga-LW01186, and Ga-LW02002. Ga-LW01158, Ga-LW01186, and Ga-LW02002 were confirmed to be GRPR antagonists by intracellular calcium release assays using PC-3 cells (Figure 3). ATP (50 nM, a positive control) and bombesin (50 nM, an agonist control) induced Ca^2+^ efflux corresponding to 222 ± 21.7 and 499 ± 73.4 relative fluorescence units (RFUs), respectively. For 50 nM of Ga-LW01158, Ga-LW01186, and Ga-LW02002, 12.6 ± 2.22, 6.64 ± 2.44, and 8.24 ± 2.28 RFUs were observed, respectively, which were significantly lower than the values of ATP and bombesin. The blank control (Dulbecco’s phosphate-buffered saline, DPBS) and the antagonist control ([D-Phe^6^,Leu-NHEt^13^,des-Met^14^]Bombesin(6-14), 50 nM) induced Ca^2+^ efflux with 7.37 ± 2.23 and 38.2 ± 7.20 RFUs, respectively.

The hydrophilicity of [^68^Ga]Ga-LW01158, [^68^Ga]Ga-LW01186, and [^68^Ga]Ga-LW02002 were determined by the shake flask method, and their LogD_7.4_ values were calculated to be −1.98 ± 0.10, −2.03 ± 0.10, and −2.33 ± 0.03, respectively (n = 3).

### 2.3. PET Imaging and Ex Vivo Biodistribution

**Figure 4:**
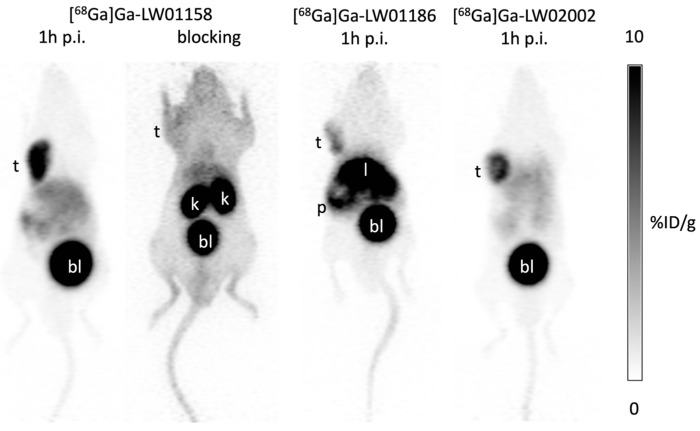
Representative PET images of [^68^Ga]Ga-LW01158, [^68^Ga]Ga-LW01186, and [^68^Ga]Ga-LW02002 acquired at 1 h post-injection in mice bearing PC-3 tumor xenografts. t: tumor; k: kidney; p: pancreas; l: liver; bl: urinary bladder.

The PC-3 tumor xenografts were clearly visualized in PET images acquired at 1 h post-injection using [^68^Ga]Ga-LW01158, [^68^Ga]Ga-LW01186, and [^68^Ga]Ga-LW02002 (Figure 4). All three tracers were primarily excreted via the renal pathway. [^68^Ga]Ga-LW01158 had the best tumor-to-background contrast among all three tracers. While [^68^Ga]Ga-LW01186 showed significant pancreas and liver uptake, the uptake in these two organs was much lower for [^68^Ga]Ga-LW01158 and [^68^Ga]Ga-LW02002. Co-injection with 100 μg of nonradioactive standard decreased the uptake of [^68^Ga]Ga-LW01158 in the PC-3 tumor xenograft to a value that was close to the background level.

**Figure 5:**
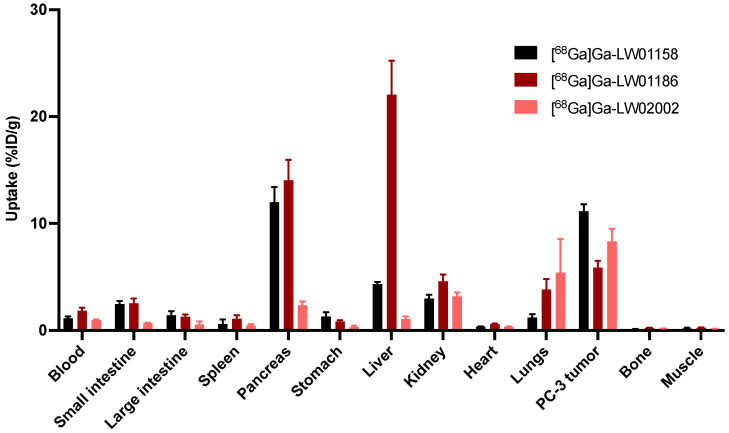
Uptake of [^68^Ga]Ga-LW01158, [^68^Ga]Ga-LW01186, and [^68^Ga]Ga-LW02002 in PC-3 tumor xenografts and major organs/tissues of NRG mice at 1 h post-injection (n = 4). Error bars indicate standard deviation.

**Figure 6:**
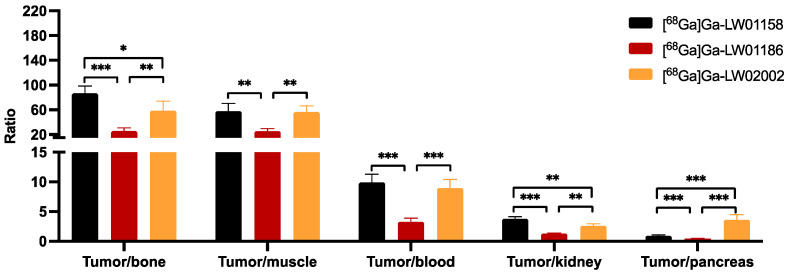
Tumor-to-organ uptake ratios of [^68^Ga]Ga-LW01158, [^68^Ga]Ga-LW01186, and [^68^Ga]Ga-02002 obtained from PC-3 tumor-bearing mice at 1 h post-injection (n = 4). Error bars indicate standard deviation. * *p* < 0.05; ** *p* < 0.01; *** *p* < 0.001.

**Figure 7:**
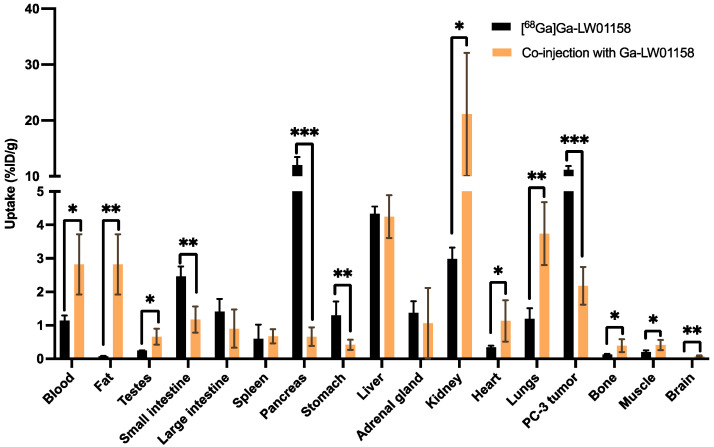
Comparison of the uptakes of [^68^Ga]Ga-LW01158 with/without co-injection of 100 µg of nonradioactive Ga-LW01158 in PC-3 tumor xenografts and major organs/tissues in mice at 1 h post-injection. Error bars indicate standard deviation (n = 4). * *p* < 0.05; ** *p* < 0.01; *** *p* <0.001.

Biodistribution studies of [^68^Ga]Ga-LW01158, [^68^Ga]Ga-LW01186, and [^68^Ga]Ga-LW02002 were also performed at 1 h post-injection in PC-3 tumor-bearing mice, and the results were consistent with the observations from their PET images (Figure 5, Figure 6 and Figure 7 and Table S4). The previously reported biodistribution data of [^68^Ga]Ga-TacsBOMB2 and [^68^Ga]Ga-RM2 obtained from the same PC-3 tumor model are also included for comparison (Table S4) [19]. Among all three TacsBOMB2-derived tracers, [^68^Ga]Ga-LW01158 had the highest tumor uptake (11.2 ± 0.65 %ID/g), which was comparable to those of [^68^Ga]Ga-TacsBOMB2 (10.2 ± 2.27 %ID/g; *p* = 0.46) and [^68^Ga]Ga-RM2 (10.5 ± 2.03%ID/g; *p* = 0.56). The tumor uptake values of [^68^Ga]Ga-LW02002 and [^68^Ga]Ga-LW01186 were 8.32 ± 1.20 and 5.87 ± 0.64%ID/g, respectively. [^68^Ga]Ga-LW01186 showed the highest pancreas uptake at 1 h post-injection (14.1 ± 1.90%ID/g), followed by [^68^Ga]Ga-LW01158 (12.0 ± 1.41%ID/g) and [^68^Ga]Ga-LW02002 (2.36 ± 0.36%ID/g). The pancreas uptake values for all three tracers were significantly lower than that of [^68^Ga]Ga-RM2 (41.9 ± 10.1%ID/g). In addition, [^68^Ga]Ga-LW01186 showed the highest liver uptake (22.1 ± 3.19%ID/g), while the liver uptake values for both [^68^Ga]Ga-LW01158 and [^68^Ga]Ga-LW02002 were only 4.33 ± 0.22 and 1.06 ± 0.24%ID/g, respectively.

Among all three ^68^Ga-labeled TacsBOMB2 derivatives, [^68^Ga]Ga-LW01158 had the highest tumor uptake and higher tumor-to-background contrast ratios for most of the major organs/tissues (Figure 6 and Table S4). [^68^Ga]Ga-LW01158 had better tumor-to-bone and tumor-to-kidney uptake ratios than [^68^Ga]Ga-LW02002 (86.6 ± 12.0 vs. 58.8 ± 15.2 and 3.76 ± 0.36 vs. 2.62 ± 0.33, respectively). However, with the lowest pancreas uptake, [^68^Ga]Ga-LW02002 had a higher tumor-to-pancreas uptake ratio than [^68^Ga]Ga-LW01158 (3.60 ± 0.86 vs. 0.94 ± 0.15; *p* < 0.001). The tumor-to-bone, tumor-to-muscle, tumor-to-blood, tumor-to-kidney, and tumor-to-pancreas uptake ratios of [^68^Ga]Ga-LW01186 were the lowest among these three tracers with values of 25.6 ± 4.94, 24.9 ± 4.63, 3.26 ± 0.59, 1.26 ± 0.10, and 0.42 ± 0.04 (*p* < 0.01), respectively.

Co-injection with 100 μg of nonradioactive standard reduced the uptake of [^68^Ga]Ga-LW01158 in PC-3 tumor xenograft by 81% (11.2 ± 0.65 to 2.18 ± 0.56%ID/g; *p* < 0.001) at 1 h post-injection. Furthermore, a significant reduction in the uptake of [^68^Ga]Ga-LW01158 was also found in the pancreas (12.0 ± 1.41 to 0.66 ± 0.28%ID/g, *p* < 0.001), small intestine (2.46 ± 0.30 to 1.17 ± 0.39%ID/g, *p* < 0.01), and stomach (1.30 ± 0.41 to 0.42 ± 0.15%ID/g; *p* < 0.01) (Figure 7 and Table S4).

### 2.4. In Vivo Stability

All [^68^Ga]Ga-LW01158, [^68^Ga]Ga-LW01186, and [^68^Ga]Ga-LW02002 showed good in vivo stability in NRG mice (n = 3, Figures S9–S11). There were 80.7 ± 1.57% of [^68^Ga]Ga-LW01158, 76.5 ± 2.91% of [^68^Ga]Ga-LW01186, and 76.6 ± 7.00% of [^68^Ga]Ga-LW02002 remaining intact in plasma at 15 min post-injection. No intact tracer was detected in urine samples for either [^68^Ga]Ga-LW01158 or [^68^Ga]Ga-LW02002, while 43.6 ± 3.46% of intact [^68^Ga]Ga-LW01186 was detected in urine samples at 15 min post-injection.

## 3. Discussion

Our group previously reported the synthesis and evaluation of a GRPR-targeted tracer, [^68^Ga]Ga-TacsBOMB2 (Figure 1A), based on a potent GRPR antagonist, RC-3950-II ([D-Phe^6^,Leu^13^ψThz^14^]Bombesin(7-14)), reported by the Schally group [17,18,19]. [^68^Ga]Ga-TacsBOMB2 showed good uptake (10.2 ± 2.27%ID/g) in PC-3 tumor xenograft and minimum pancreas uptake (2.81 ± 0.78%ID/g) at 1 h post-injection [19]. In this study, we modified the GRPR-targeting sequence of [^68^Ga]Ga-TacsBOMB2 with unnatural amino acid substitutions and evaluated the potential of the resulting ligands for PET imaging. Recently, our group systematically substituted the amino acids (Gln^7^, Trp^8^, Ala^9^, Val^10^, Gly^11^, and His^12^) at potential cleavage sites of a previously reported GRPR agonist tracer ([^68^Ga]Ga-TacBOMB2: [^68^Ga]Ga-DOTA-Pip-D-Phe^6^-Gln^7^-Trp^8^-Ala^9^-Val^10^-Gly^11^-His^12^-Leu^13^-Thz^14^-NH_2_) with unnatural amino acids to improve in vivo stability [23]. We identified that Tle^10^ and NMe-His^12^ substitutions significantly improved in vivo stability and retained good binding affinity, high PC-3 tumor uptake, and minimal pancreas uptake [23]. Therefore, in this study, we replaced Val^10^ and His^12^ in [^68^Ga]Ga-TacsBOMB2 with Tle^10^ and NMe-His^12^, respectively, and evaluated the potential of the resulting Ga-LW01158 (Figure 1B) and Ga-LW01160 (Figure 1C), respectively, for GRPR targeting.

We first determined the binding affinities of Ga-LW01158 and Ga-LW01160 using an in vitro competition binding assay (Figure 2). The K_i_ value of Ga-LW01158 was 5.11 ± 0.47 nM, which was better than that of Ga-TacsBOMB2 (7.08 ± 0.65 nM) [19]. This observation is consistent with our previous finding showing that Tle^10^ substitution on the GRPR agonist Ga-TacBOMB2 improves binding affinity [23]. However, Ga-LW01160 showed very poor binding toward GRPR (K_i_ = 187 ± 17.8 nM), while the previously reported NMe-His^12^ substitution significantly improved the binding affinity of Ga-TacBOMB2 from 7.62 ± 0.19 nM to 2.98 ± 0.69 nM [23,24]. These data demonstrate that Tle^10^ substitution is tolerable in both GRPR agonists and antagonists, while NMe-His^12^ substitution can only be applied to GRPR agonists without significantly reducing the binding affinity. One possible explanation for this observation is that GRPR agonists and antagonists might bind to the receptors in different configurations so that modifications to some specific amino acids are tolerable only by either antagonists or agonists.

Next, we introduced an additional αMe-Trp^8^ substitution to LW01158 to obtain LW01186 (Figure 1D). αMe-Trp^8^ substitution has been successfully used by the Wester group for the design of the potent and in vivo-stable GRPR-targeted antagonist AMTG, derived from RM2 [25]. NMe-Gly^11^ substitution has also been reported for the design of GRPR-targeted ligands used to improve in vivo stability [26,27]. Previously, we developed a GRPR antagonist, Ga-TacsBOMB5, by introducing the NMe-Gly^11^ substitution to Ga-TacsBOMB2 [19]. Though [^68^Ga]Ga-TacsBOMB5 was not metabolically more stable than [^68^Ga]Ga-TacsBOMB2, it had a better PC-3 tumor uptake and tumor-to-background imaging contrast than [^68^Ga]Ga-TacsBOMB2 at 1 h post-injection [19]. Therefore, in this study, we also combined NMe-Gly^11^ and Tle^10^ substitutions to generate Ga-LW02002 (Figure 1E). As expected, good GRPR binding affinities for both Ga-LW01186 and Ga-LW02002 were observed (K_i_ = 6.94 ± 0.95 and 11.0 ± 0.39 nM, respectively) (Figure 2). These data also support that the configurations of GRPR binding with agonists and antagonists might be different. While αMe-Trp^8^ and NMe-Gly^11^ substitutions are tolerable in antagonists, we previously showed that αMe-Trp^8^ and NMe-Gly^11^ substitutions significantly reduce the binding affinity of GRPR agonists.

The GRPR antagonist characteristics of the three potent Ga-TacsBOMB2 derivatives were determined using in vitro intracellular calcium release assays (Figure 3). In comparison with the positive control (ATP) and agonist control (bombesin), Ga-LW01158, Ga-LW01186, and Ga-LW02002 induced significantly lower intracellular Ca^2+^ efflux. This indicates that Tle^10^ substitution in Ga-TacsBOMB2, either alone or in combination with αMe-Trp^8^ or NMe-Gly^11^ substitution, retains antagonist characteristics.

Imaging studies showed that the PC-3 tumor xenograft could be clearly visualized in PET images using all three ^68^Ga-labeled tracers ([^68^Ga]Ga-LW01158, [^68^Ga]Ga-LW01186, and [^68^Ga]Ga-LW02002), confirming their good in vivo GRPR-targeting capabilities. All three ^68^Ga-labeled tracers were mainly excreted via the renal pathway owing to the hydrophilic nature of these tracers (LogD_7.4_ values ≤ −1.98). The ex vivo biodistribution data of [^68^Ga]Ga-LW01158, [^68^Ga]Ga-LW01186, and [^68^Ga]Ga-LW02002 were consistent with the findings in their PET images (Figure 4, Figure 5, Figure 6 and Figure 7 and Table S4). Among these [^68^Ga]Ga-TacsBOMB2 derivatives, [^68^Ga]Ga-LW01158 had the highest PC-3 tumor uptake (11.2 ± 0.65%ID/g) compared with 5.87 ± 0.64%ID/g for [^68^Ga]Ga-LW01186 and 8.32 ± 1.20%ID/g for [^68^Ga]Ga-LW02002. This might have resulted from the fact that Ga-LW01158 has a better GRPR binding affinity than Ga-LW01186 and Ga-LW02002 (K_i_ = 5.11 ± 0.47, 6.94 ± 0.95, and 11.0 ± 0.39 nM, respectively). This also indicates that, by using the Ga-TacsBOMB2 pharmacophore, the combination of αMe-Trp^8^ or NMe-Gly^11^ substitution with Tle^10^ substitution cannot further improve either the binding affinity to GRPR or increase the uptake in GRPR-expressing PC-3 tumor xenografts.

[^68^Ga]Ga-LW02002 had lower uptake values in the liver, small intestine, and large intestine (1.06 ± 0.24, 0.66 ± 0.06, and 0.54 ± 0.29%ID/g, respectively) than those of [^68^Ga]Ga-LW01158 and [^68^Ga]Ga-LW01186. This is consistent with its relatively higher hydrophilicity than the other two tracers (LogD_7.4_ = −2.33 ± 0.03 vs. −1.98 ± 0.10 for [^68^Ga]Ga-LW01158 and −2.03 ± 0.10 for [^68^Ga]Ga-LW01186). Based on the LogD_7.4_ value of [^68^Ga]Ga-LW01186 (−2.03 ± 0.10), its high liver uptake (22.1 ± 3.19%ID/g) was unexpected. Although the cause of its high liver uptake remains to be investigated, the high liver uptake could be one of the reasons leading to its lower uptake in PC-3 tumor xenografts (5.87 ± 0.64%ID/g) when compared with [^68^Ga]Ga-LW01158 (11.2 ± 0.65%ID/g) and [^68^Ga]Ga-LW02002 (8.32 ± 1.20%ID/g).

Compared with the previously reported biodistribution data of [^68^Ga]Ga-RM2 (Table S4) [19], all three [^68^Ga]Ga-TacsBOMB2 derivatives showed significantly lower uptake in the pancreas. This is consistent with our previous finding showing that [D-Phe^6^,Leu^13^ψThz^14^]Bombesin(6-14) is a promising pharmacophore for the design of GRPR-targeted radiopharmaceuticals with a minimal pancreas uptake. One possible explanation is that these three [^68^Ga]Ga-TacsBOMB2 derivatives are more selective for binding to the human GRPR expressed in PC-3 tumors in comparison with the mouse GRPR expressed in mouse pancreas. The low pancreas uptake of these three [^68^Ga]Ga-TacsBOMB2 derivatives also demonstrates that αMe-Trp^8^, NMe-Gly^11^, and Tle^10^ substitutions do not significantly increase the pancreas uptake of the resulting GRPR-targeted tracers. With a significantly lower uptake in the pancreas and a comparable tumor uptake compared with the clinically validated [^68^Ga]Ga-RM2, [^68^Ga]Ga-LW01158 is a promising radiopharmaceutical for detecting GRPR-expressing lesions with PET, especially for lesions in or adjacent to the pancreas. Similarly, LW01158 might be promising for labeling with ^177^Lu for radioligand therapy to minimize toxicity to the pancreas.

A blocking study (Figure 7 and Table S4) was conducted to tease out the specificity of our top candidate, [^68^Ga]Ga-LW01158. The uptake in GRPR-expressing PC-3 tumor xenografts was reduced by >80% with the co-injection of 100 µg of nonradioactive standard, confirming the tumor uptake of [^68^Ga]Ga-LW01158 is specific. Moreover, significant reductions were also observed in the pancreas (12.0 ± 1.41 to 0.66 ± 0.28%ID/g; *p* < 0.001), stomach (1.30 ± 0.41 to 0.42 ± 0.15 %ID/g, *p* < 0.01), and small intestine (2.46 ± 0.30 to 1.17 ± 0.39%ID/g, *p* < 0.01). This is in agreement with the physiological expression pattern of GRPR in normal tissue/organs [1]. In addition, a significantly increased uptake was observed in kidneys (2.98 ± 0.34 to 21.1 ± 11.0%ID/g; *p* < 0.01). This is most likely due to the competitive binding of the nonradioactive standard to the GRPR in PC-3 tumors, increasing the amount of free [^68^Ga]Ga-LW01158 to be metabolized and excreted via the renal pathway. Furthermore, GRPR-targeted ligands are mainly metabolized by NEP, which is highly expressed in kidneys [20,21]. Co-injection with a significant amount of nonradioactive standard could saturate the metabolism of [^68^Ga]Ga-LW01158 caused by NEP in kidneys, leading to higher kidney absorption and the retention of [^68^Ga]Ga-LW01158.

In vivo stability studies revealed that all three [^68^Ga]Ga-TacsBOMB2 derivatives were relatively stable in vivo with 76.5 to 80.7% of the tracer remaining intact in mouse plasma at 15 min post-injection. These values were comparable to that of the previously reported [^68^Ga]Ga-TacsBOMB2 (83.3 ± 1.45%) [19]. This suggests that, among the potential cleavage sites on the [^68^Ga]Ga-TacsBOMB2 pharmacophore for peptidases, the amide bond between His^12^-Leu^13^ is the major one. Since the amide bond between His^12^-Leu^13^ was already stabilized by the introduction of a reduced peptide bond (Leu^13^ψThz^14^), no further improvements in in vivo stability were observed with the additional Tle^10^ substitution, either alone or in combination with αMe-Trp^8^ or NMe-Gly^11^ substitution.

No intact tracer was detected in urine samples of [^68^Ga]Ga-LW01158, [^68^Ga]Ga-LW02002, and the previously reported [^68^Ga]Ga-TacsBOMB2 at 15 min post-injection. Interestingly, although [^68^Ga]Ga-LW01186 had a similar intact fraction in mouse plasma when compared with [^68^Ga]Ga-LW01158, [^68^Ga]Ga-LW02002, and [^68^Ga]Ga-TacsBOMB2, 43.6 ± 3.46% of intact [^68^Ga]Ga-LW01186 was detected in urine samples at 15 min post-injection (Figure S10). This observation was consistent with a recent report by the Wester group showing that the αMe-Trp^8^ substitution in [^177^Lu]Lu-RM2 significantly increased the intact fraction of the resulting [^177^Lu]Lu-AMTG in urine samples at 30 min post-injection (0.5 ± 0.1% to 68.2 ± 3.1%) [25]. This suggests that αMe-Trp^8^ substitution greatly inhibits the degradation of GRPR-targeted ligands by peptidases expressed in the kidneys.

## 4. Materials and Methods

### 4.1. General Methods

Fmoc-LeuψThz-OH hydrochloride was synthesized following our previously published procedures [19]. All other chemicals and solvents were purchased from commercial sources and used without further purification. GRPR-targeted peptides were synthesized on solid phase using an AAPPTec (Louisville, KY, USA) Endeavor 90 peptide synthesizer. Purification and quality control of DOTA-conjugated peptides and their ^nat^Ga/^68^Ga-complexed analogs were conducted on Agilent (Santa Clara, CA, USA) HPLC systems equipped with a model 1200 quaternary pump, a model 1200 UV absorbance detector (220 nm), and a Bioscan (Washington, DC, USA) NaI scintillation detector. The operation of Agilent HPLC systems was controlled using the Agilent ChemStation software (Version A.01.05 (1.3.19.115)). A semi-preparative column (Luna C18; 5 µm; 250 × 10 mm) and an analytical column (Luna C18; 5 µm; 250 × 4.6 mm) purchased from Phenomenex (Torrance, CA, USA) were used for purification and quality control, respectively. The HPLC eluates were collected and lyophilized with a Labconco (Kansas City, MO, USA) FreeZone 4.5 Plus freeze-drier. MS analyses of DOTA-conjugated peptides and their ^nat^Ga-complexed analogs were performed with a Waters (Milford, MA, USA) Acquity QDa mass spectrometer equipped with a 2489 UV/Vis detector and an e2695 Separations module. C18 Sep-Pak cartridges (1 cm^3^, 50 mg) were purchased from Waters. ^68^Ga was eluted from an ITM Medical Isotopes GmbH (Munich, Germany) generator and purified according to previously published procedures using a DGA resin column from Eichrom Technologies LLC (Lisle, IL, USA) [28]. The radioactivity of ^68^Ga-labeled peptides was measured using a Capintec (Ramsey, NJ, USA) CRC^®^-25R/W dose calibrator. The radioactivity measurements for samples collected from biodistribution studies, binding assays, in vivo stability tests, and LogD_7.4_ assays were counted using a Perkin Elmer (Waltham, MA, USA) Wizard2 2480 automatic gamma counter.

### 4.2. Synthesis of DOTA-Conjugated Peptides

LW01158, LW01160, LW01186, and LW02002 were synthesized on solid phase using Fmoc peptide chemistry. Sieber resin (0.05 mmol) was treated with 20% piperidine in *N*,*N*-dimethylformamide (DMF) to remove the Fmoc-protecting group. Fmoc-LeuψThz-OH (5 eq.), Fmoc-protected amino acids (5 eq.), and Fmoc-4-amino-1-carboxymethyl-piperidine (5 eq.) were pre-activated with HATU (5 eq.), HOAt (5 eq.) and *N*,*N*-diisopropylethylamine (DIEA, 15 eq.) and coupled to the resin sequentially. DOTA(*t*Bu)_3_ (5 eq.) pre-activated with HATU (5 eq.) and DIEA (25 eq.) was coupled to the resin at the *N*-terminus.

For cleavage and simultaneously removing protecting groups, the resin was treated with a cocktail mixture of trifluoroacetic acid (TFA, 81.5%), triisopropylsilane (TIS 1.0%), water (5%), 2,2′-(ethylenedioxy)diethanethiol (DODT, 2.5%), thioanisole (5%), and phenol (5%) at room temperature for 4 h. The cleaved peptides were filtrated and then precipitated by cold diethyl ether. The crude peptides were collected by centrifugation and purified by HPLC (semi-preparative column). The eluates containing the desired peptides were collected and lyophilized. The HPLC conditions, retention times, isolated yields, and MS confirmations of DOTA-conjugated peptides are provided in Table S1 and Figures S1–S4.

### 4.3. Synthesis of Nonradioactive Ga-Complexed Standards

The nonradioactive Ga-complexed standards were synthesized by incubating the DOTA-conjugated precursor (1 eq.) and GaCl_3_ (1.0 M; 5 eq.) in NaOAc buffer (0.1 M; 500 µL; pH 4.5) at 80 °C for 15 min. The reaction mixture was then purified with HPLC (semi-preparative column). The HPLC eluates containing the desired peptide were collected and lyophilized. The HPLC conditions, retention times, isolated yields, and MS confirmations of the nonradioactive Ga-complexed standards are provided in Table S2 and Figures S5–S8.

### 4.4. Synthesis of ^68^Ga-Labeled Tracers

The radiolabeling experiments were performed following previously published procedures [28,29,30]. Purified ^68^GaCl_3_ in 0.5 mL of water was added to a vial preloaded with 0.7 mL of HEPES buffer (2 M, pH 5.0) and 10 μL of precursor solution (1 mM). The radiolabeling reaction was conducted by 100 °C microwave heating for 1 min (Monowave 200, Anton Paar, Graz, Austria) followed by HPLC purification using the semi-preparative column. The eluate fraction containing the radiolabeled product was collected, diluted with water (50 mL), and passed through a C18 Sep-Pak cartridge that was pre-washed with ethanol (1 mL) and water (2 mL). The ^68^Ga-labeled product was eluted off the cartridge with ethanol (0.4 mL) containing 1% ascorbic acid and diluted with PBS containing 1% ascorbic acid for imaging and biodistribution studies. Quality control was performed with HPLC on the analytical column. The HPLC conditions and retention times for purification and quality control are provided in Table S3.

### 4.5. LogD_7.4_ Measurement

The LogD_7.4_ values of [^68^Ga]Ga-LW01158, [^68^Ga]Ga-LW01186, and [^68^Ga]Ga-LW02002 were measured using the shake flask method following previously published procedures [28]. Briefly, an aliquot of ^68^Ga-labeled peptide was added to a 15 mL falcon tube containing a mixture of n-octanol (3 mL) and DPBS (3 mL; 0.1 M; pH 7.4). The mixture was vortexed for 1 min followed by centrifugation at 3000 rpm for 15 min. Samples of the n-octanol (1 mL) and buffer (1 mL) layers were collected and measured in a gamma counter. LogD_7.4_ was calculated with the following equation: LogD_7.4_ = log_10_[(counts in the n-octanol phase)/(counts in the buffer phase)].

### 4.6. Cell Culture

Known to overexpress GRPR, the PC-3 cell line, a human prostate cancer cell line, has been widely used for the in vitro and in vivo evaluation of GRPR-targeted ligands for decades [4,8]. Thus, our group chose the PC-3 cell line for this study. The PC-3 cells obtained from ATCC (via Cedarlane, Burlington, Canada) were cultured in RPMI 1640 medium (Life Technologies Corporations, Carlsbad, CA, USA) supplemented with 10% FBS, penicillin (100 U/mL) and streptomycin (100 μg/mL) at 37 °C in a Panasonic Healthcare (Tokyo, Japan) MCO-19AIC humidified incubator containing 5% CO_2_. The cells were confirmed to be pathogen-free via the IMPACT Rodent Pathogen Test (IDEXX BioAnalytics, Columbia, MO, USA). Cells grown to 80–90% confluence were washed with sterile DPBS (pH 7.4) and collected after 1 min trypsinization at 37 °C. The cell concentration was measured in duplicate using a Moxi mini automated cell counter (ORFLO Technologies, Ketchum, ID, USA).

### 4.7. Fluorometric Calcium Release Assay

Following previously published procedures [31,32], 5 × 10^4^ PC-3 cells in 100 μL of growth media were seeded per well in a 96-well clear-bottom black plate 24 h prior to the assay. A loading buffer (100 μL/well) containing a calcium-sensitive dye (FLIPR Calcium 6 assay kit) was added to the 96-well plate. After incubation at 37 °C for 2 h, the plate was placed in a FlexStation 3 microplate reader (Molecular Devices, San Jose, CA, USA). TacsBOMB2 derivatives (50 nM), [D-Phe^6^,Leu-NHEt^13^,des-Met^14^]Bombesin(6-14) (50 nM, antagonist control), bombesin (50 nM, agonist control), adenosine triphosphate (ATP, 50 nM, positive control), or DPBS (blank control) was added, and the fluorescent signals were acquired for 2 min (λ_Ex_ = 485 nm; λ_Em_ = 525 nm; n = 3). The relative fluorescent units (RFUs = max − min) were measured to determine the agonistic/antagonistic properties.

### 4.8. In Vitro Competition Binding Assay

Inhibition constants (K_i_) of GRPR-targeted ligands were measured by in vitro competition binding assay using PC-3 cells and [^125^I-Tyr^4^]Bombesin as the radioligand. PC-3 cells were seeded in 24-well poly-D-lysine plates at 2 × 10^5^ cells/well 48 h prior to the assay. The growth medium was replaced with 400 μL of reaction medium (RPMI 1640 containing 2 mg/mL of BSA and 20 mM of HEPES). After 1 h incubation at 37 °C. Ga-LW01158, Ga-LW01160, Ga-LW01186, and Ga-LW02002 in 50 μL of reaction medium with decreasing concentrations (10 μM to 1 pM) and 50 μL of 0.01 nM [^125^I-Tyr^4^]Bombesin were added to the wells followed by incubation with moderate agitation for 1 h at 37 °C. Cells were gently washed with ice-cold PBS twice, harvested via trypsinization, and counted for radioactivity on a Perkin Elmer (Waltham, MA, USA) Wizard2 2480 automatic gamma counter. Data were analyzed using nonlinear regression (one binding site model for competition assay) with the GraphPad (San Diego, CA, USA) Prism 8 software (Version 8.4.3).

### 4.9. Ex Vivo Biodistribution, PET/CT Imaging, and In Vivo Stability Studies

PET/CT imaging, biodistribution, and in vivo stability studies were conducted using male NOD.Cg-Rag1^tm1Mom^ Il2rg^tm1Wjl^/SzJ (NRG) mice following previously published procedures [28,31,32,33]. The experiments were conducted according to the guidelines established by the Canadian Council on Animal Care and approved by the Animal Ethics Committee of the University of British Columbia. The mice were anesthetized through inhalation of 2.5% isoflurane in 2 mL/min oxygen and implanted subcutaneously with 5 × 10^6^ PC-3 cells (100 µL; 1:1 PBS:Matrigel) behind the left shoulder. Mice were used for PET/CT imaging and biodistribution studies when the tumor grew to 5–8 mm in diameter over around 4 weeks.

PET/CT imaging experiments were performed on a Siemens (Knoxville, TN, USA) Inveon micro-PET/CT scanner. The tumor-bearing mice were injected with 3–5 MBq of ^68^Ga-labeled tracer through a lateral caudal tail vein under anesthesia, followed by recovery and roaming freely in their cages during the uptake period. At 50 min post-injection, a 10 min CT scan was conducted first for localization and attenuation correction after segmentation to reconstruct the PET images, followed by a 10 min static PET imaging acquisition.

For biodistribution studies, the mice were injected with the radiotracer (2–4 MBq) via the tail vein as described above. For blocking, the mice were co-injected with [^68^Ga]Ga-LW01158 and 100 μg of its nonradioactive standard. At 1 h post-injection, the mice were anesthetized via isoflurane inhalation and euthanized via CO_2_ inhalation. Blood was collected through cardiac puncture, and organs/tissues of interest were collected, weighed, and counted using a Perkin Elmer (Waltham, MA, USA) Wizard2 2480 automatic gamma counter.

For in vivo stability studies, 5–13 MBq of [^68^Ga]Ga-LW01158, [^68^Ga]Ga-LW01186, or [^68^Ga]Ga -LW02002 was injected via a lateral caudal tail vein into healthy male NRG mice (n = 3). At 15 min post-injection, the urine and blood samples were collected after the mice were anesthetized and euthanized. The plasma was extracted from whole blood by adding CH_3_CN (500 μL), 1 min of vortex, centrifugation, and the separation of supernatant. The plasma and urine samples were analyzed via radio-HPLC by using the conditions for the quality control of these ^68^Ga-labeled radioligands (Table S3).

### 4.10. Statistical Analysis

Statistical analyses were performed with Student’s *t*-test using the Microsoft (Redmond, WA, USA) Excel software (Version 16.84 (24041420)). A comparison of biodistribution data between two tracers was conducted using an unpaired two-tailed test. The unpaired one-tailed test was used to compare the biodistribution data of [^68^Ga]Ga-LW01158 with/without co-injection of nonradioactive Ga-LW01158. A statistically significant difference was considered when the adjusted *p*-value was <0.05.

## 5. Conclusions

The Tle^10^ substitution, either alone or in combination with αMe-Trp^8^ or NMe-Gly^11^, in the GRPR binding sequence of Ga-TacsBOMB2 generates derivatives that retained good GRPR binding affinity, antagonist characteristics, and good in vivo stability. However, the substitution of His^12^ with NMe-His leads to a significant decrease in GRPR binding affinity. In comparison with the clinically validated [^68^Ga]Ga-RM2, [^68^Ga]Ga-LW01158 has comparable tumor uptake but much less pancreas uptake. Therefore, [^68^Ga]Ga-LW01158 is promising for clinical development for detecting GRPR-expressing lesions with PET, particularly for lesions in or adjacent to the pancreas. With a superior tumor-to-pancreas uptake ratio, [^68^Ga]Ga-LW02002 might be more promising for detecting cancer lesions adjacent to and in the pancreas.

## 6. Patents

The compounds disclosed in this report are covered by a recent patent application (PCT/CA2023/050401; filing date: 23 March 2023). Lei Wang, Zhengxing Zhang, Chengcheng Zhang, François Bénard, and Kuo-Shyan Lin are listed as inventors in this filed patent application.

## Data Availability

The data presented in this study are available in the Supplementary Materials.

## Supplementary Materials

The following supporting information can be downloaded at https://www.mdpi.com/article/10.3390/ph17050621/s1: Table S1: MS characterizations, yields, and HPLC purification conditions of LW01158, LW01160, LW01186, and LW02002. Table S2: MS characterizations, yields, and HPLC purification conditions of Ga-LW01158, Ga-LW01160, Ga-LW01186, and Ga-LW02002. Table S3: HPLC conditions for the purification and quality control of ^68^Ga-labeled LW01158, LW01186, and LW02002. Table S4: Biodistribution and uptake ratios of ^68^Ga-labeled GRPR-targeted tracers in PC-3 tumor-bearing mice. Figure S1: The MS spectrum of LW01158. Figure S2: The MS spectrum of LW01160. Figure S3: The MS spectrum of LW01186. Figure S4: The MS spectrum of LW02002. Figure S5: The MS spectrum of Ga-LW01158. Figure S6: The MS spectrum of Ga-LW01160. Figure S7: The MS spectrum of Ga-LW01186. Figure S8: The MS spectrum of Ga-LW02002. Figure S9: Representative radio-HPLC chromatograms from analysis of an intact fraction of [^68^Ga]Ga-LW01158 in mouse plasma and urine samples. Figure S10: Representative radio-HPLC chromatograms from analysis of an intact fraction of [^68^Ga]Ga-LW01186 in mouse plasma and urine samples. Figure S11: Representative radio-HPLC chromatograms from analysis of an intact fraction of [^68^Ga]Ga-LW02002 in mouse plasma and urine samples.
