# Supplementary material for: Synthesis and Evaluation of Novel 68Ga-Labeled [D-Phe6,Leu13ψThz14]bombesin(6-14) Analogs for Cancer Imaging with Positron Emission Tomography
Source: Pharmaceuticals (Basel). 2024 May 11;17(5):621. doi: 10.3390/ph17050621 (PMC11124507; doi:10.3390/ph17050621)
Supplement: Supplementary file 1 [file pharmaceuticals-17-00621-s001.zip › pharmaceuticals-2977952-SI.pdf]

SUPPLEMENTAL INFORMATION

Table S1: MS characterizations, yields and HPLC purification conditions of LW01158, LW01160, LW01186, and LW02002.

Compound name	HPLC conditions	Retention time (min)	Yield (%)	Calculated mass (m/z)	Found (m/z)
LW01158	26% CH_3CN and 0.1% TFA in H_2O; flow rate 4.5 mL/min	14.1	32	$[M+2H]^{2+}$ 799.4	$[M+2H]^{2+}$ 799.6
LW01160	23% CH_3CN and 0.1% TFA in H_2O; flow rate 4.5 mL/min	10.6	24	$[M+2H]^{2+}$ 799.4	$[M+2H]^{2+}$ 1599.2
LW01186	29% CH_3CN and 0.1% TFA in H_2O; flow rate 4.5 mL/min	12.1	8	$[M+2H]^{2+}$ 806.4	$[M+2H]^{2+}$ 806.8
LW02002	27% CH_3CN and 0.1% TFA in H_2O; flow rate 4.5 mL/min	14.5	17	$[M+2H]^{2+}$ 806.4	$[M+2H]^{2+}$ 806.7

Table S2: MS characterizations, yields and HPLC purification conditions of Ga-LW01158, Ga-LW01160, Ga-LW01186, and Ga-LW02002.

Compound name	HPLC conditions	Retention time (min)	Yield (%)	Calculated mass (m/z)	Found (m/z)
Ga-LW01158	26% CH_3CN and 0.1% TFA in H_2O; flow rate 4.5 mL/min	13.2	76	$[M+2H]^{2+}$ 832.9	$[M+2H]^{2+}$ 833.4
Ga-LW01160	25% CH_3CN and 0.1% TFA in H_2O; flow rate 4.5 mL/min	9.3	81	$[M+2H]^{2+}$ 832.9	$[M+2H]^{2+}$ 833.6
Ga-LW01186	29% CH_3CN and 0.1% TFA in H_2O; flow rate 4.5 mL/min	13.5	81	$[M+2H]^{2+}$ 839.9	$[M+2H]^{2+}$ 839.5
Ga-LW02002	27% CH_3CN and 0.1% TFA in H_2O; flow rate 4.5 mL/min	16.3	77	$[M+2H]^{2+}$ 839.9	$[M+2H]^{2+}$ 839.4

Table S3: HPLC conditions for the purification and quality control of ^{68}Ga-labeled LW01158, LW01186, and LW02002. FA: formic acid.

Compound name	HPLC conditions		Retention time (min)
$[^{68}Ga]$Ga-LW01158	Semi-Prep	20% CH_3CN and 0.1% FA in H_2O; flow rate 4.5 mL/min	12.5
	QC	21.5% CH_3CN and 0.1% FA in H_2O; flow rate 2.0 mL/min	6.1
$[^{68}Ga]$Ga-LW01186	Semi-Prep	22% CH_3CN and 0.1% FA in H_2O; flow rate 4.5 mL/min	11.6
	QC	24% CH_3CN and 0.1% FA in H_2O; flow rate 2 mL/min	5.5
$[^{68}Ga]$Ga-LW02002	Semi-Prep	20% CH_3CN and 0.1% FA in H_2O; flow rate 4.5 mL/min	14.3
	QC	21.5% CH_3CN and 0.1% FA in H_2O; flow rate 2.0 mL/min	6.7

Table S4: Biodistribution (mean ± SD, n = 4) and uptake ratios of ^{68}Ga-labeled GRPR-targeted tracers in PC-3 tumor-bearing mice. The biodistribution data of [^{68}Ga]Ga-TacsBOMB2 and [^{68}Ga]Ga-RM2 have been reported previously [1], and are included here for comparison. The mice in the blocked group were co-injected with 100 µg of Ga-LW01158. *, **, and *** indicate p < 0.05, <0.01 and < 0.001, respectively, when comparing the 1 h and 1 h blocked data of [^{68}Ga]Ga-LW01158.

Tissue (%ID/g)	[^{68}Ga]Ga-TacsBOMB2 1 h	[^{68}Ga]Ga-LW01158 1 h	[^{68}Ga]Ga-LW01158 1 h blocked	[^{68}Ga]Ga-LW01186 1 h	[^{68}Ga]Ga-LW02002 1 h	[^{68}Ga]Ga-RM2 1 h
Blood	0.76 ± 0.21	1.14 ± 0.15	2.82 ± 0.90*	1.83 ± 0.31	0.94 ± 0.08	0.64 ± 0.10
Fat	0.09 ± 0.03	0.07 ± 0.01	0.27 ± 0.10**	0.16 ± 0.03	0.07 ± 0.01	0.05 ± 0.03
Testes	0.19 ± 0.05	0.24 ± 0.01	0.66 ± 0.24*	0.31 ± 0.05	0.20 ± 0.10	0.18 ± 0.03
Small intestine	1.04 ± 0.30	2.46 ± 0.30	1.17 ± 0.39**	2.54 ± 0.44	0.66 ± 0.06	5.08 ± 1.05
Large intestine	0.37 ± 0.16	1.41 ± 0.38	0.90 ± 0.57	1.27 ± 0.21	0.54 ± 0.29	2.19 ± 0.67
Spleen	0.47 ± 0.17	0.60 ± 0.42	0.67 ± 0.21	1.09 ± 0.33	0.46 ± 0.13	0.44 ± 0.26
**Pancreas**	**2.81 ± 0.78**	**12.0 ± 1.41**	**0.66 ± 0.28*** **	**14.1 ± 1.90**	**2.36 ± 0.36**	**41.9 ± 10.1**
Stomach	0.32 ± 0.08	1.30 ± 0.41	0.42 ± 0.15**	0.83 ± 0.13	0.32 ± 0.11	3.87 ± 2.80
Liver	2.61 ± 0.70	4.33 ± 0.22	4.25 ± 0.64	22.1± 3.19	1.06 ± 0.24	0.84 ± 0.55
Adrenal glands	0.57 ± 0.40	1.37 ± 0.35	1.06 ± 1.06	2.75 ± 0.74	0.58 ± 0.07	3.01 ± 0.91
Kidneys	2.51 ± 0.59	2.98 ± 0.34	21.1 ± 11.0*	4.58 ± 0.66	3.19 ± 0.36	2.57 ± 0.48
Heart	0.27 ± 0.08	0.34 ± 0.05	1.13 ± 0.62*	0.56 ± 0.09	0.33 ± 0.06	0.19 ± 0.03
Lungs	0.75 ± 0.52	1.19 ± 0.32	3.74 ± 0.94**	3.82 ± 1.00	5.42 ± 3.13	0.62 ± 0.26
**PC-3 tumor**	**10.2 ± 2.27**	**11.2 ± 0.65**	**2.18 ± 0.56*** **	**5.87 ± 0.64**	**8.32 ± 1.20**	**10.5 ± 2.03**
Bone	0.19 ± 0.06	0.13 ± 0.01	0.39 ± 0.19*	0.23 ± 0.02	0.15 ± 0.03	0.11 ± 0.03
Muscle	0.15 ± 0.05	0.20 ± 0.05	0.41 ± 0.15*	0.24 ± 0.04	0.15 ± 0.02	0.14 ± 0.06
Brain	0.05 ± 0.03	0.03 ± 0.00	0.08 ± 0.02**	0.06 ± 0.01	0.06 ± 0.03	0.03 ± 0.01
Tumor/bone	61.3 ± 25.0	86.6 ± 12.0	6.18 ± 1.67***	25.6 ± 4.94	58.8 ± 15.2	96.5 ± 27.1
Tumor/muscle	70.1 ± 14.2	58.0 ± 12.5	5.53 ± 0.84***	24.9 ± 4.63	56.4 ± 9.92	80.8 ± 27.5
Tumor/blood	14.0 ± 3.48	9.91 ± 1.38	0.79 ± 0.11***	3.26 ± 0.59	8.93 ± 1.49	16.5 ± 3.06
Tumor/kidney	4.10 ± 0.46	3.76 ± 0.36	0.11 ± 0.03***	1.26 ± 0.10	2.62 ± 0.33	4.13 ± 0.73
Tumor/pancreas	3.70 ± 0.55	0.94 ± 0.15	3.43 ± 0.58***	0.42 ± 0.04	3.60 ± 0.86	0.25 ± 0.04

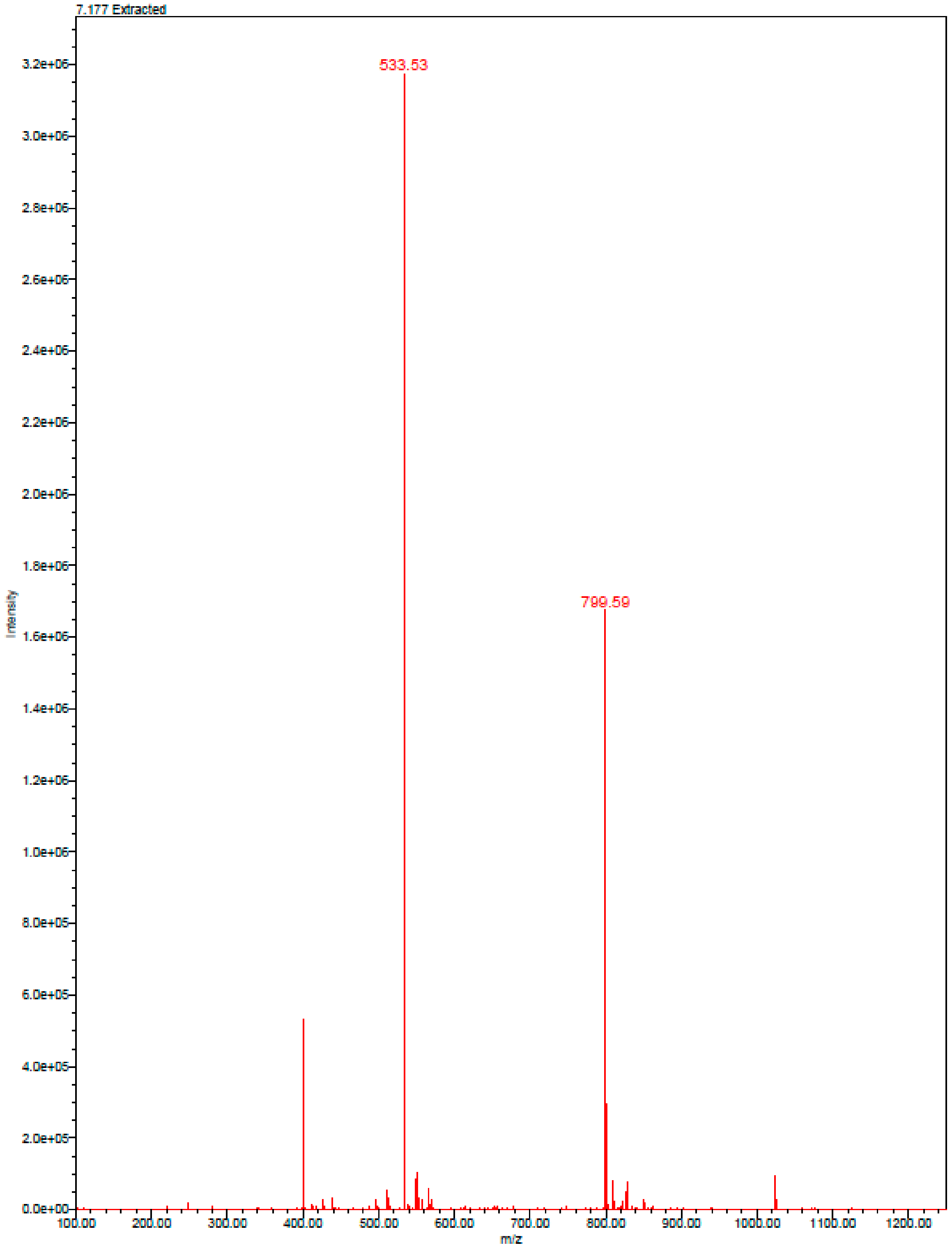

Figure S1: The MS spectrum of LW01158: calculated $[M+2H]^{2+}$ (m/z) 799.4; found 799.6.

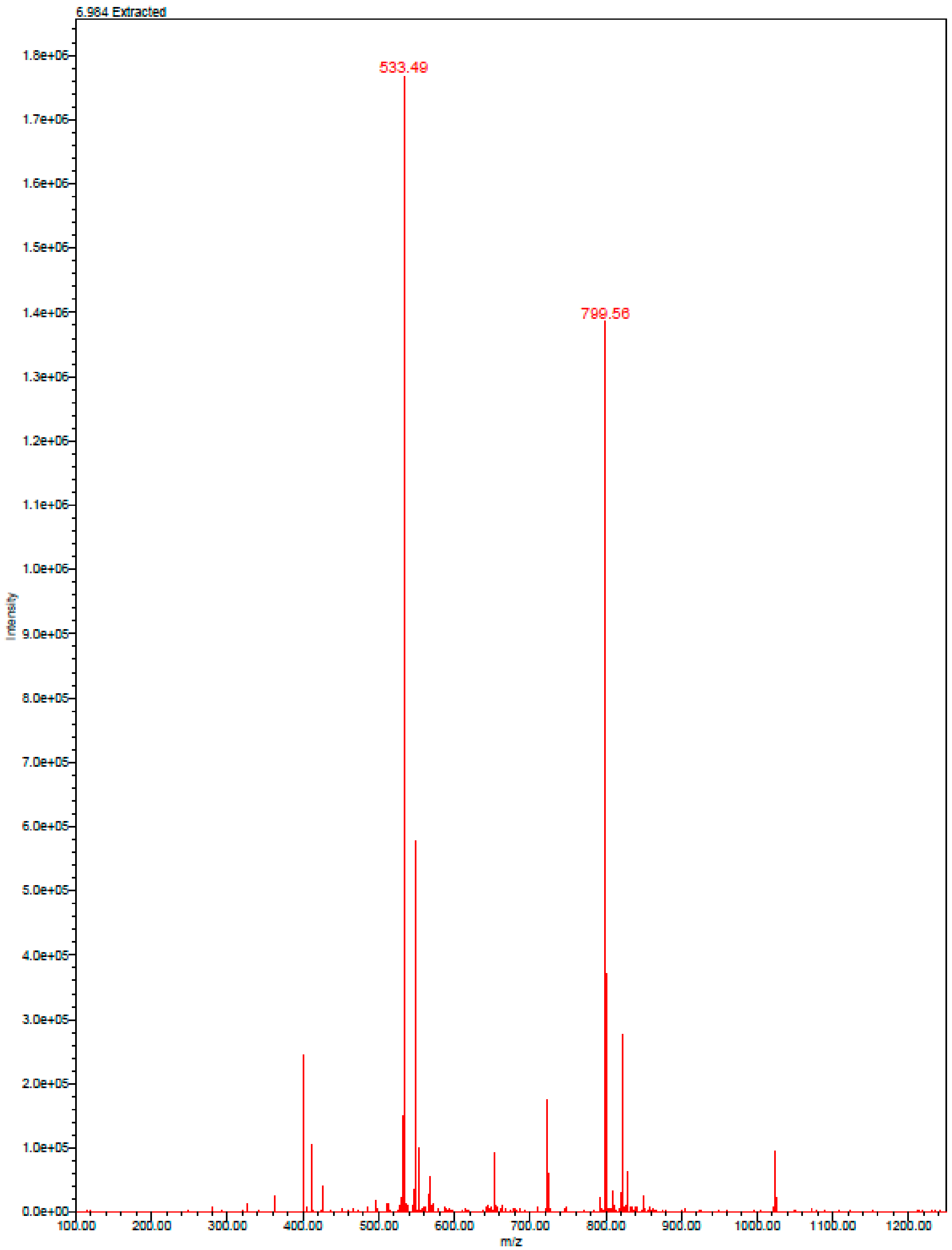

Figure S2: The MS spectrum of LW01160: calculated $[M+2H]^{2+}$ (m/z) 799.4; found 799.6.

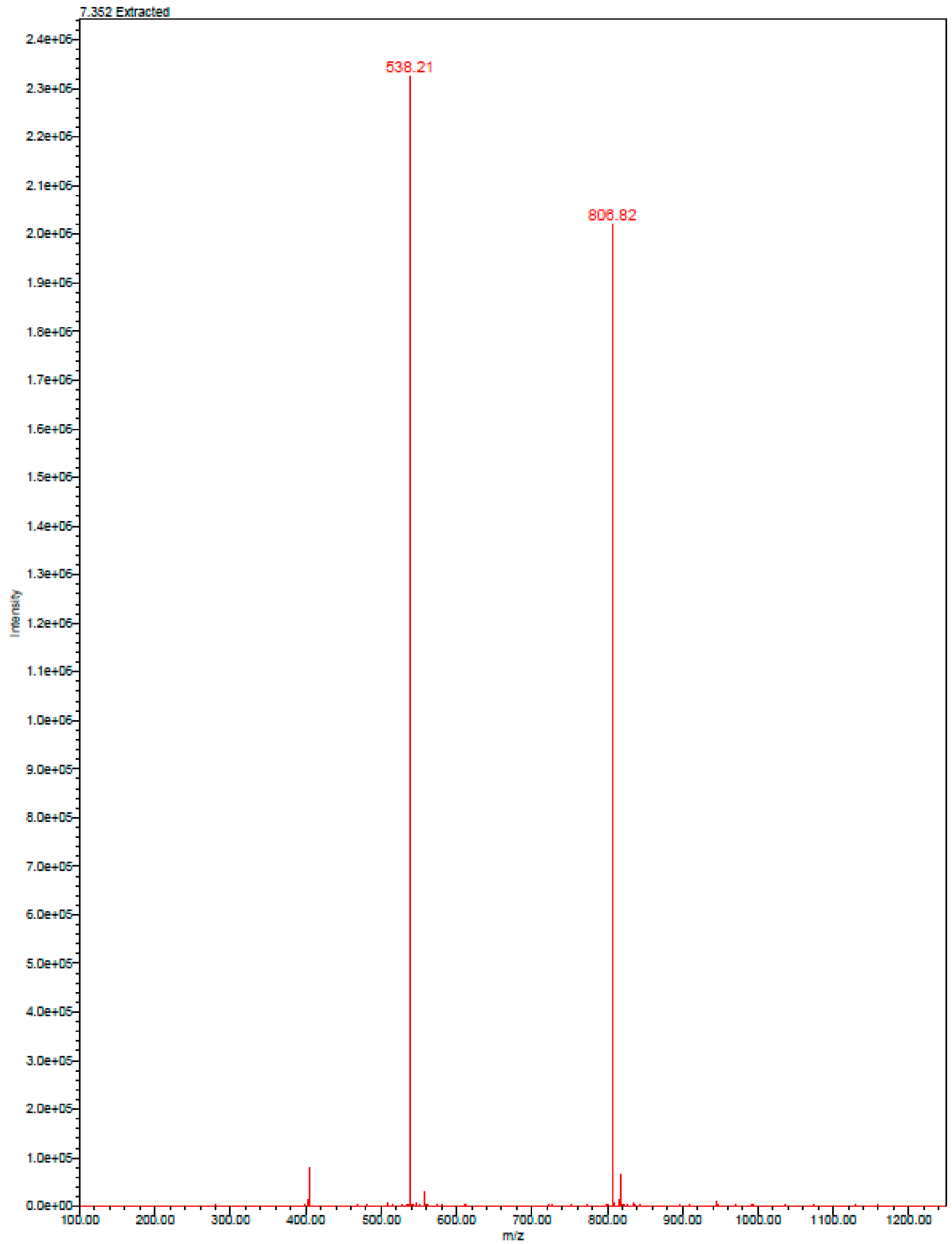

Figure S3: The MS spectrum of LW01186: calculated $[M+2H]^{2+}$ (m/z) 806.4; found 806.8.

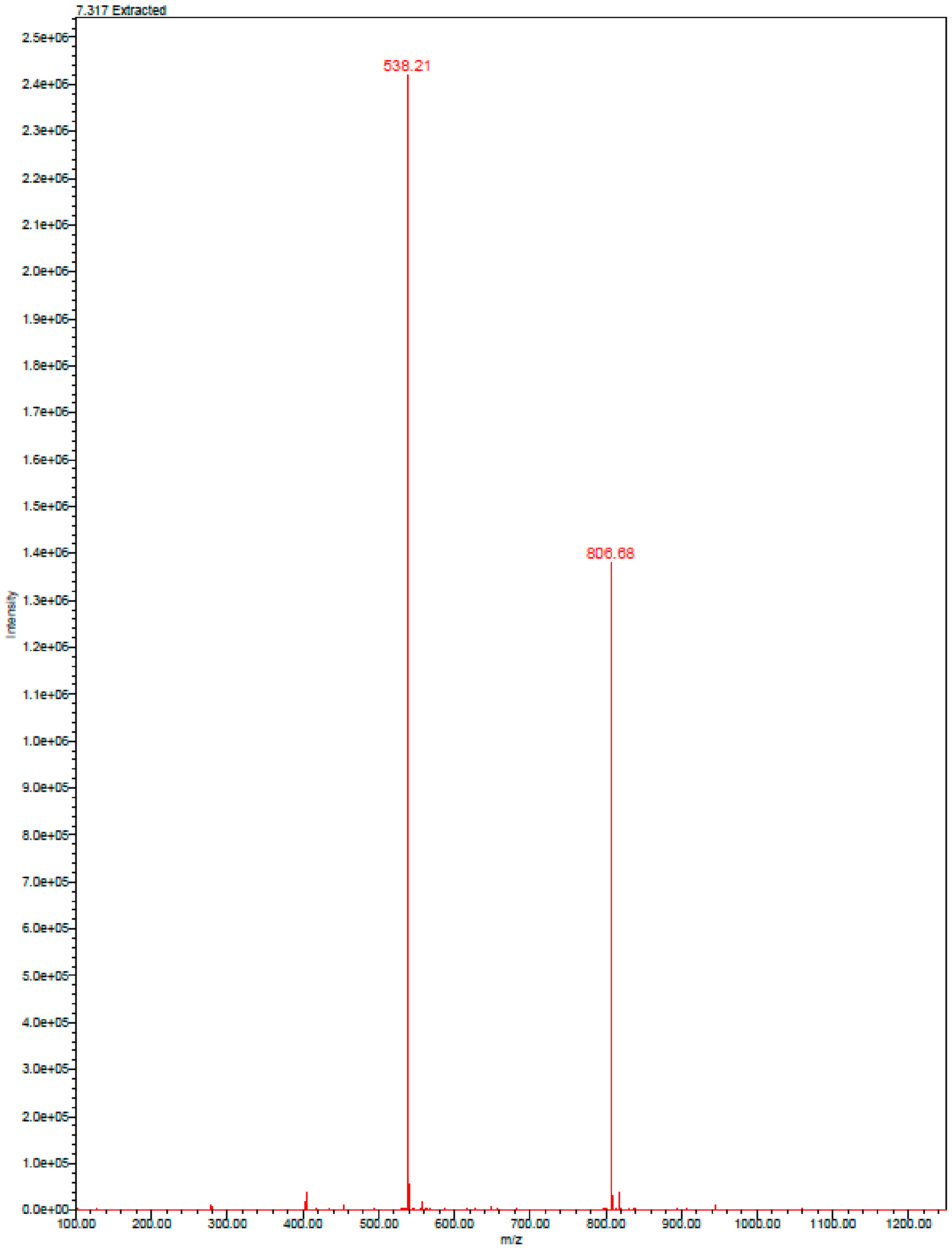

Figure S4: The MS spectrum of LW02002: calculated $[M+2H]^{2+}$ (m/z) 806.4; found 806.7.

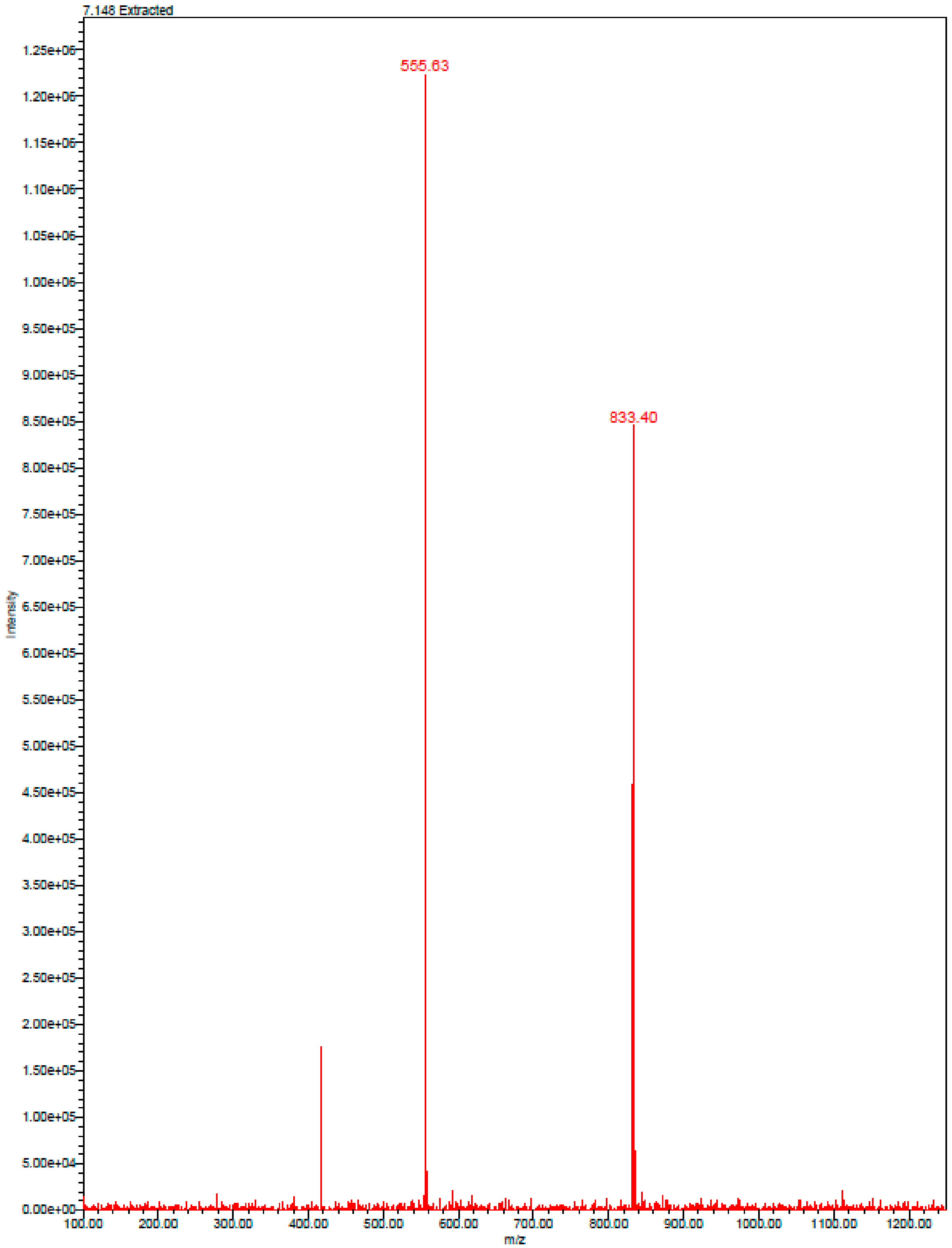

Figure S5: The MS spectrum of Ga-LW01158: calculated $[M+2H]^{2+}$ (m/z) 832.9; found 833.4.

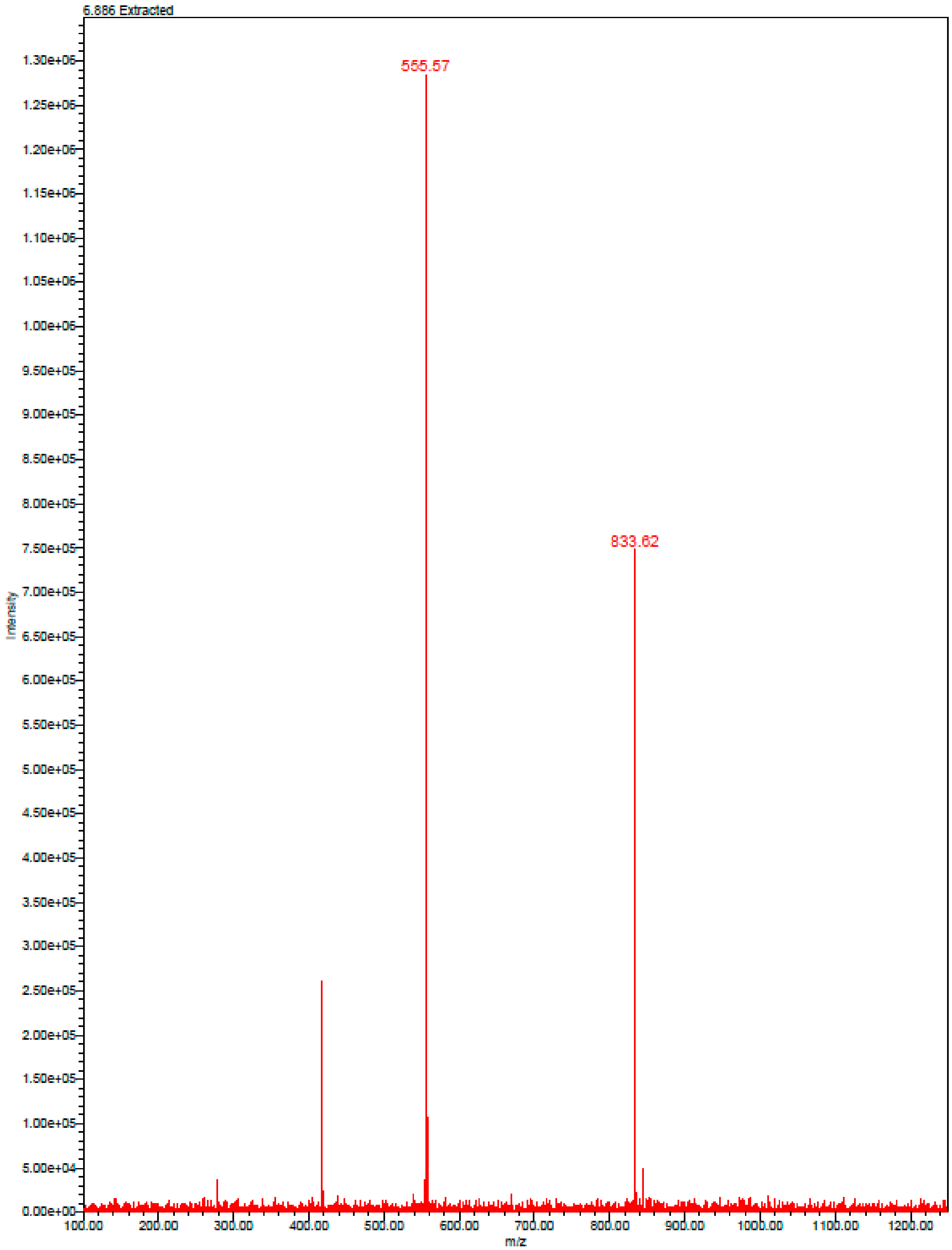

Figure S6: The MS spectrum of Ga-LW01160: calculated $[M+2H]^{2+}$ (m/z) 832.9; found 833.6.

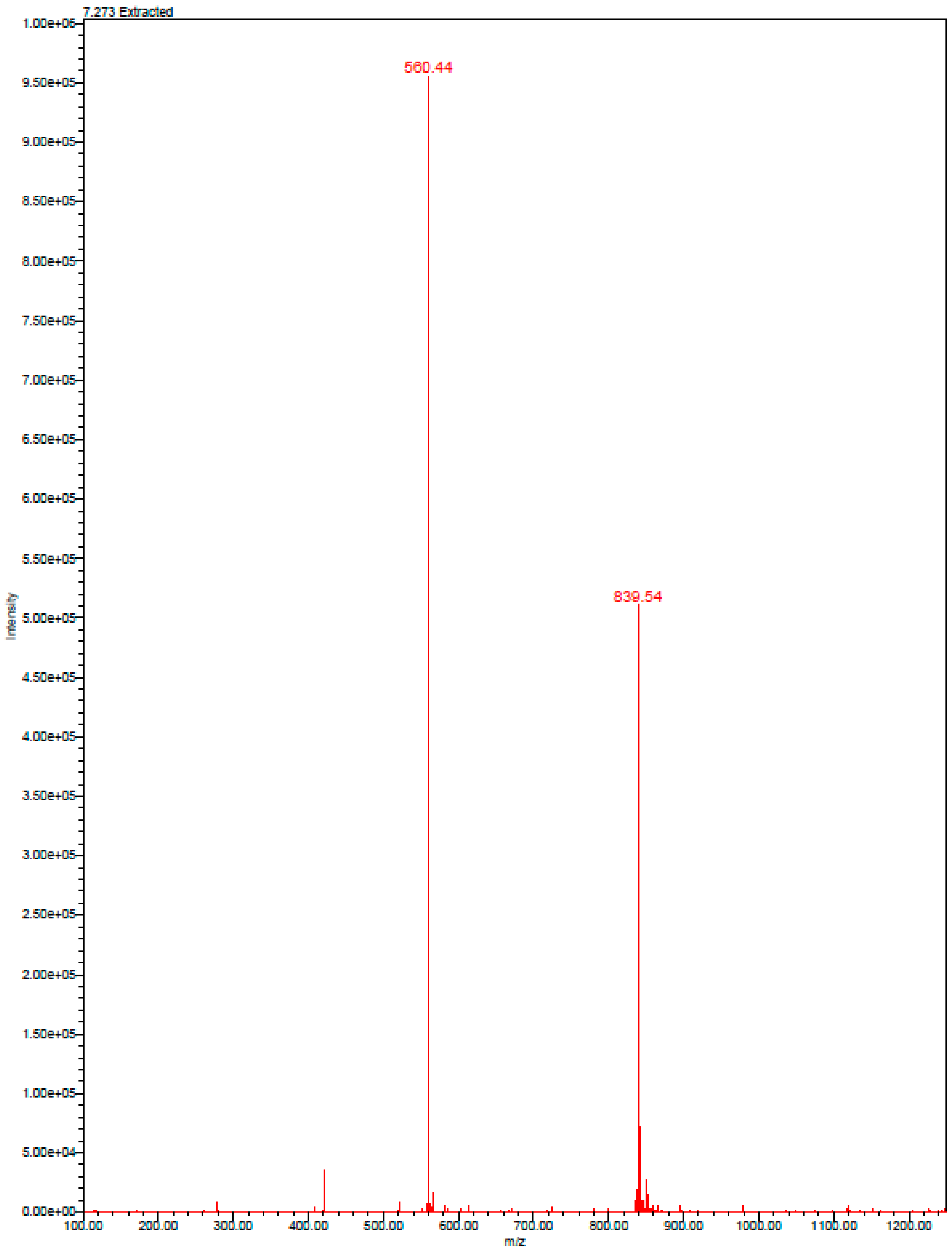

Figure S7: The MS spectrum of Ga-LW01186: calculated $[M+2H]^{2+}$ (m/z) 839.9; found 839.5.

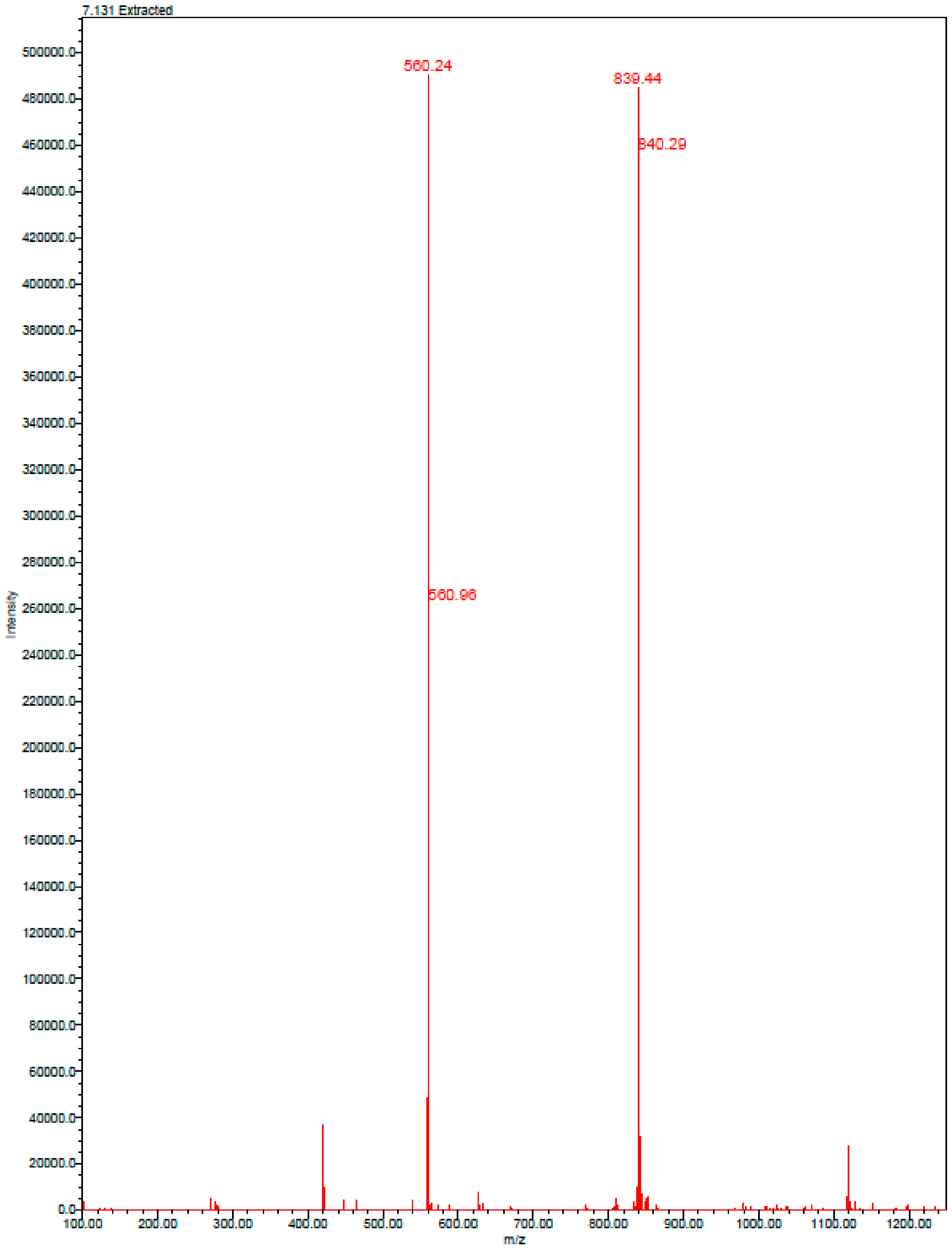

Figure S8: The MS spectrum of Ga-LW02002: calculated $[M+2H]^{2+}$ (m/z) 839.9; found 839.4.

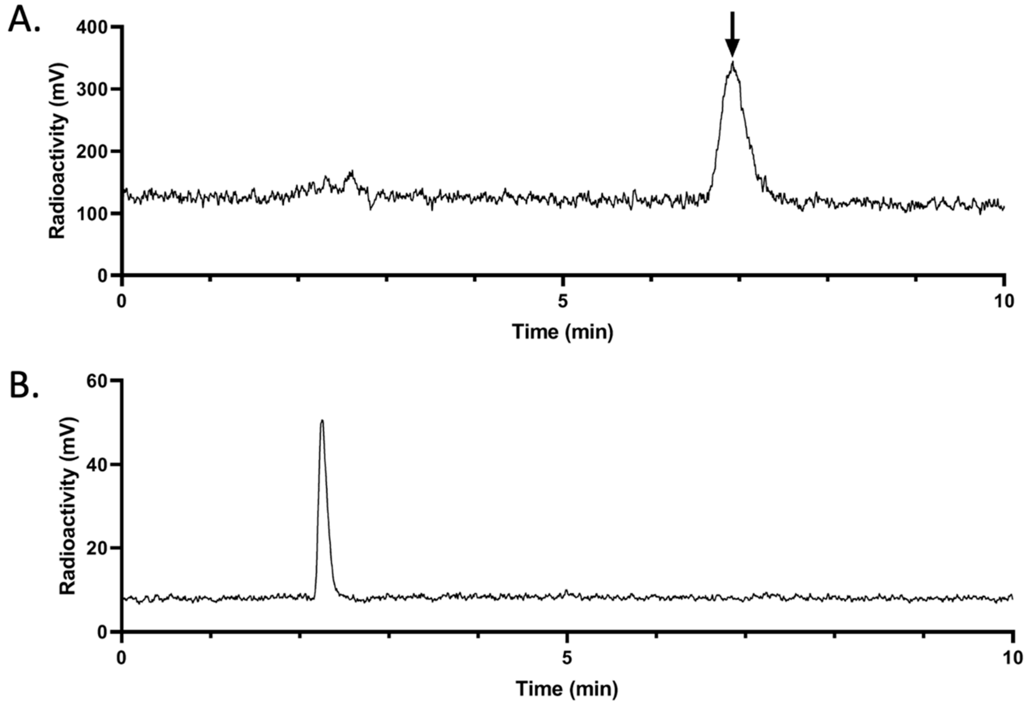

Figure S9: Representative radio-HPLC chromatograms from analysis of intact fraction of [^{68}Ga]Ga-LW01158 in (A) mouse plasma and (B) urine samples collected at 15 min post-injection. The peak pointed by an arrow is the intact tracer.

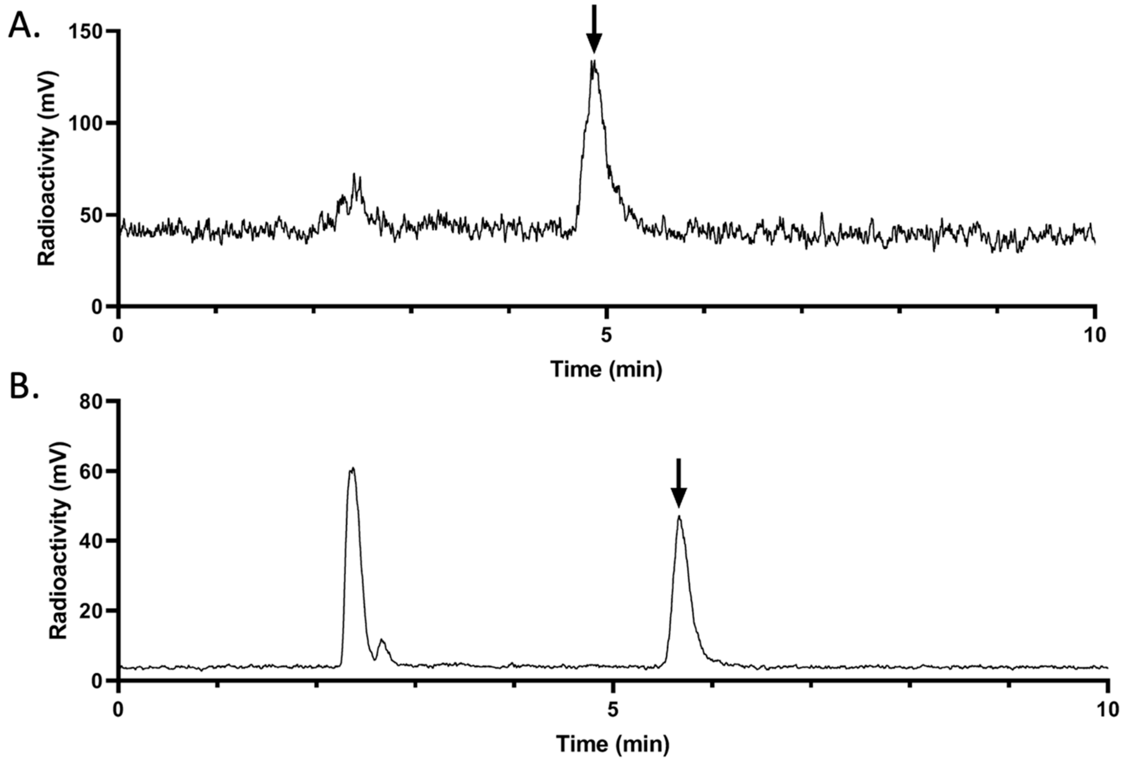

Figure S10: Representative radio-HPLC chromatograms from analysis of intact fraction of [^{68}Ga]Ga-LW01186 in (A) mouse plasma and (B) urine samples collected at 15 min post-injection. The peak pointed by an arrow is the intact tracer.

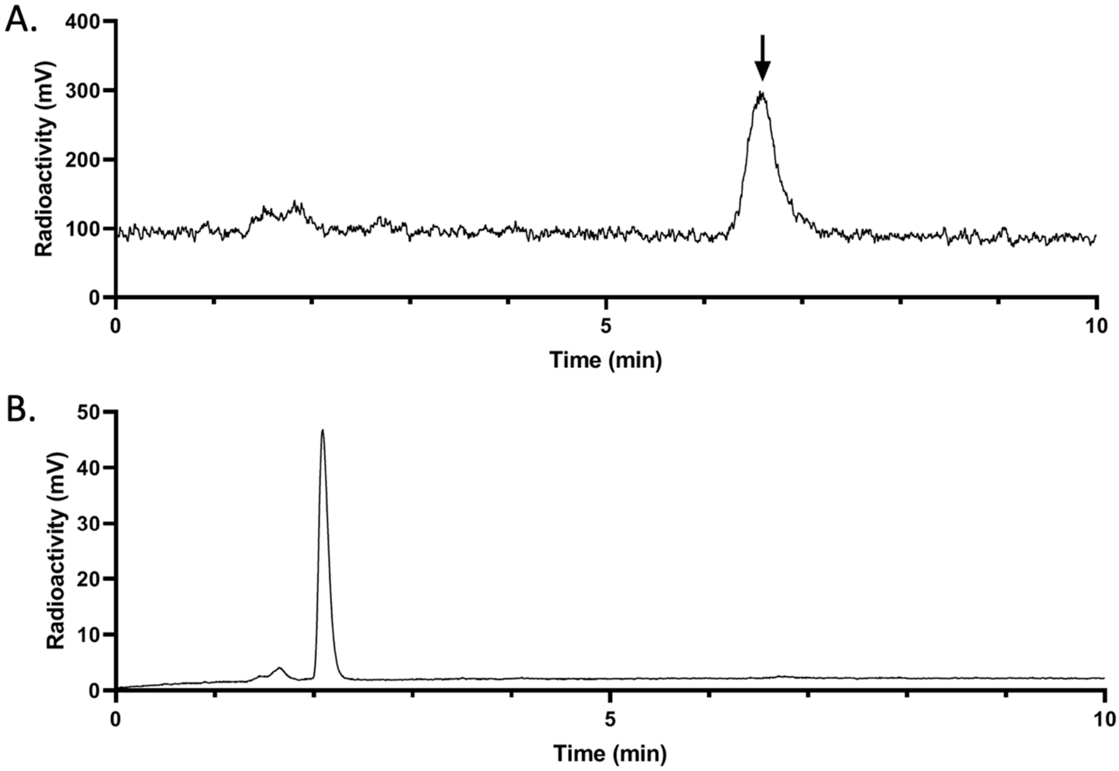

Figure S11: Representative radio-HPLC chromatograms from analysis of intact fraction of [^{68}Ga]Ga-LW02002 in (A) mouse plasma and (B) urine samples collected at 15 min post-injection. The peak pointed by an arrow is the intact tracer.

Reference:

1. Wang, L.; Zhang, Z.; Merkens, H.; Zeisler, J.; Zhang, C.; Roxin, A.; Tan, R.; Bénard, F.; Lin, K.-S., ^{68}Ga-Labeled [Leu^{13}ψThz^{14}]Bombesin(7–14) derivatives: Promising GRPR-targeting PET tracers with low pancreas uptake. *Molecules.* **2022**, *27*, 3777.
